# New Delhi Metallo-Beta-Lactamase Facilitates the Emergence of Cefiderocol Resistance in Enterobacter cloacae

**DOI:** 10.1128/aac.02011-21

**Published:** 2022-02-15

**Authors:** Dennis Nurjadi, Kaan Kocer, Quan Chanthalangsy, Sabrina Klein, Klaus Heeg, Sébastien Boutin

**Affiliations:** a Department of Infectious Diseases, Medical Microbiology and Hospital Hygiene, University Hospital Heidelberg, Heidelberg, Germany; b Translational Lung Research Center Heidelberg (TLRC), German Center for Lung Research (DZL), University of Heidelberg, Heidelberg, Germany

**Keywords:** *Enterobacter cloacae*, *Enterobacterales*, antibiotic resistance, carbapenem resistance, cefiderocol, cefiderocol resistance, drug resistance mechanisms, New Delhi metallo-β-lactamase

## Abstract

Cefiderocol is a promising novel siderophore cephalosporin for the treatment of multidrug-resistant Gram-negative bacilli and with stability against degradation by metallo-β-lactamases. Nonetheless, the emergence of cefiderocol in metallo-β-lactamase-producing Enterobacterales during therapy has been reported on more than one occasion. To understand the underlying mechanisms and factors facilitating the resistance development, we conducted an *in vitro* evolution experiment using clinical E. cloacae isolates via serial passaging under cefiderocol pressure. In this study, we showed that the presence of the New Delhi metallo-β-lactamase (NDM) facilitates the emergence of resistance via nonsynonymous mutations of the CirA catecholate siderophore receptor. Inhibition of metallo-β-lactamase activity using dipicolinic acid prevented the emergence of cefiderocol-resistant mutants successfully. This finding implies that caution should be taken when using cefiderocol for the treatment of infections caused by metallo-β-lactamase-producing bacteria.

## INTRODUCTION

The emergence and rapid spread of antimicrobial resistance in clinically relevant bacteria have become a major global concern (1–4). The dynamics of resistance emergence are much faster than the discovery of new antibiotics (5). One of the major clinical concerns is the acquisition of potent β-lactamase enzymes (carbapenemases), capable of inactivating almost all β-lactam antibiotics, which is one of the most important classes of antibiotics in clinical use today (2). Cefiderocol is a novel siderophore-conjugated cephalosporin antibiotic that can utilize the bacterial iron transport system to enter the periplasmatic compartment and is regarded as a stable against metallo-β-lactamases (6). Cefiderocol resistance is considered rare (7, 8).

From the clinical point of view, cefiderocol is an important and promising drug due to its stability against metallo-β-lactamase-producing bacteria (9). To date, this antibiotic substance is not yet widely in clinical use, so that knowledge on the resistance mechanisms and the circumstances under which resistances can emerge is still limited (10). In a previous clinical case on *in vivo* emergence of cefiderocol resistance in Enterobacter cloacae during therapy, we demonstrated that, despite heterogeneity (deletion, insertion, and transposon insertion), the frameshift mutations in the cefiderocol-resistant E. cloacae isolates always targeted the *cirA* gene (11). The selection for *cirA* inactivation in E. cloacae suggested the keystone function of this gene in the mode of action of cefiderocol. Most importantly, divergent evolution seemed to be the driver behind the heterogeneity of genotypic resistance. The idea of parallel or diverging evolution in the development of antibiotic resistance was not completely novel, but most studies are performed *in vitro* (12, 13), and, thus, clinical relevance may be limited. We hypothesized that antibiotic pressure due to the presence of metallo-β-lactamases facilitated the development of resistance through divergent evolution from a single clone. As a secondary objective, we aimed to verify the significance of *cirA* mutations in offering cefiderocol resistance *in vitro*.

## RESULTS

**FIG 1:**
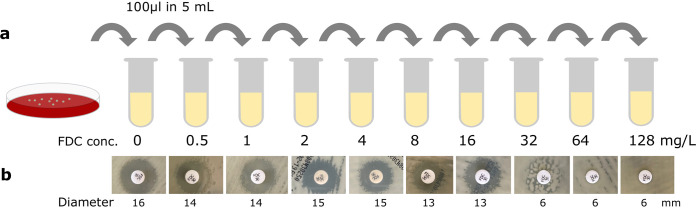
Emergence of cefiderocol resistance in serial passaging under cefiderocol pressure. (a) Serial passaging of clinical E. cloacae in increasing cefiderocol concentrations (ranged from 0.5 to 128 mg/liter) in cation-adjusted Mueller-Hinton broth (CA-MHB). After initial growth in liquid culture without antibiotics, E. cloacae were transferred to fresh CA-MHB in a 1:50 dilution factor containing cefiderocol and repeated after overnight incubation at 37°C with increasing cefiderocol concentrations. (b) Disk diffusion of liquid culture revealed the emergence of smaller colonies in the zone of inhibition after reaching cefiderocol concentrations >8 mg/liter. In high cefiderocol concentrations (>64 mg/liter), no zone of inhibition was visible indicating the development of high-level resistance toward this substance. Variations in colony color were caused by various lighting conditions. The zone of inhibition of the disk diffusion was provided in millimeters. FDC=cefiderocol.

To investigate whether we could validate the *in vivo* finding, we performed a serial passaging experiment using the clinical cefiderocol-susceptible isolate (etcl_1) from our clinical case. Subcultures (1:50) were performed daily from an initial liquid culture in cation-adjusted Mueller-Hinton broth (CA-MHB) while continually doubling the antibiotic concentration in the liquid media until either no growth was observed or a final concentration of 128 mg/liter was reached (concentrations ranged from 0.5 to 128 mg/liter). After each subculturing step, the changes in the phenotypic susceptibility were determined by performing disk diffusion using the cefiderocol-impregnated disk on standard Mueller-Hinton agar (Fig. 1a). Morphological changes were observed in the form of hazy borders of the zone of inhibition starting from a cefiderocol concentration of 4 mg/liter, which became more apparent after subculture in 8 mg/liter cefiderocol. Starting from an antibiotic concentration of 16 mg/liter and going to a concentration of 32 mg/liter, small colonies within the zone of inhibition appeared, suggesting the emergence of resistant mutants and heterogeneity in the phenotypic resistance of the bacterial population. At a concentration of 64 mg/liter, the zone of inhibition completely disappeared, indicating the dominance of phenotypically resistant mutants (Fig. 1b).

**FIG 2:**
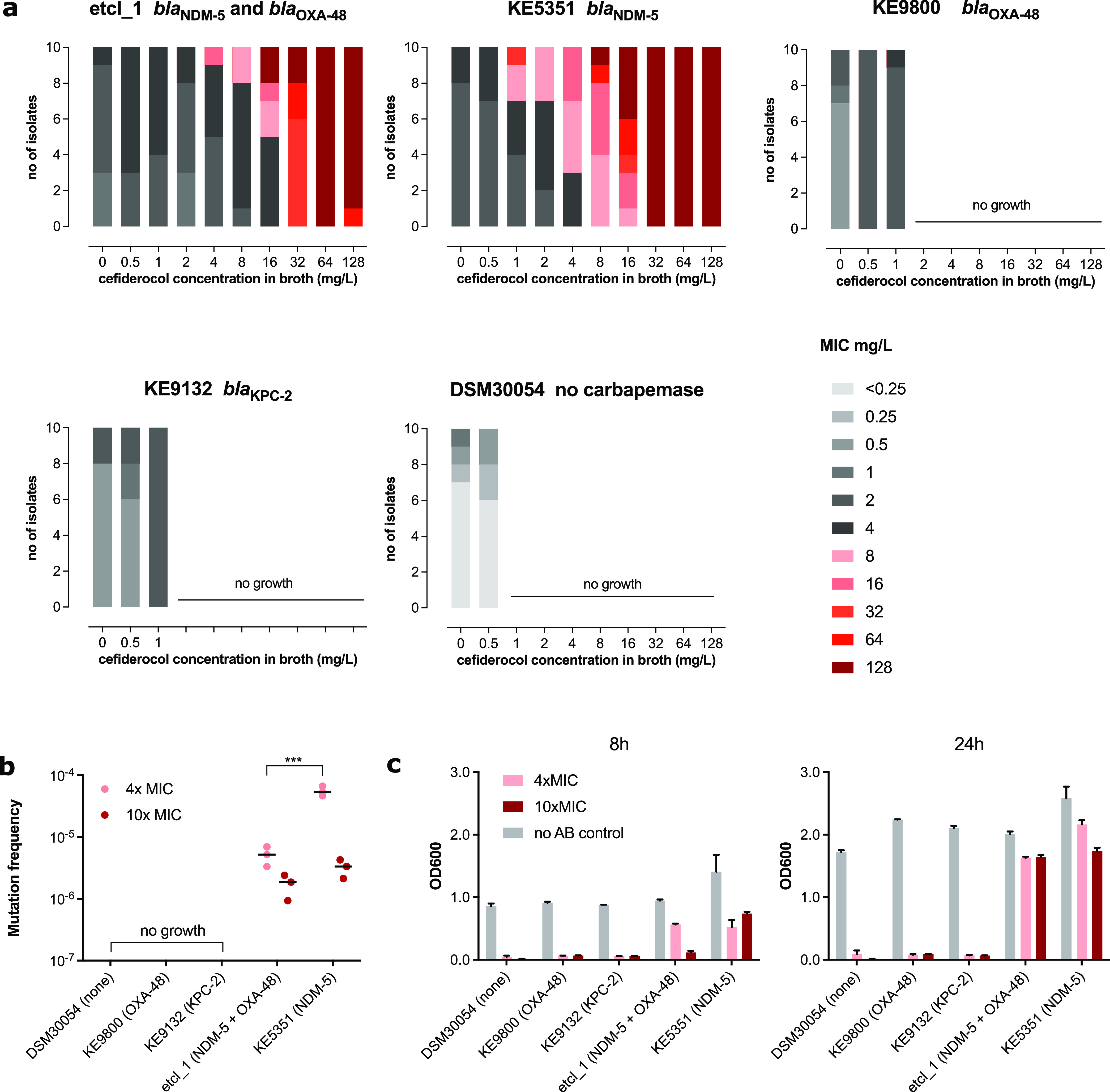
Variability of MICs and mutation frequency. (a) MICs of 10 randomly selected E. cloacae isolates growing in various cefiderocol concentrations (ranged 0.5 to 128 mg/liter) in a serial passage experimental revealed the development of heterogeneous phenotypic resistance toward cefiderocol. Bars in shades of gray indicate phenotypic susceptibility and bars in shades of red indicate MIC >4 mg/liter. (b) Mutation frequency at 4× and 10× the MIC for etcl_1 and KE5351. (c) Growth under antibiotic pressure (4× and 10× MIC) was measured by optical density at 600 nm (OD_600_). Data presented for (b) and (c) were collective data from 3 independent experiments. Where applicable, statistical analysis was performed using a two-way ANOVA. ***, *P* < 0.001.

To further study the uniformity of cefiderocol susceptibility after each subculturing process under continuous and increasing antibiotic pressure, 10 μL of the bacterial suspension from each subculture was inoculated onto a universal growth medium (Columbia blood agar) and 10 distinct colonies were picked at random for AST by broth microdilution in guideline-conform iron-depleted CA-MHB. Consistent with the results of the serial passaging, we observed the emergence of resistant (MIC >4 mg/liter) isolates starting from the subculture step at a cefiderocol concentration of 4 mg/liter, which gained dominance after subsequent subcultures in increasing cefiderocol concentrations. Gradually, the proportion of resistant isolates increased along with the MIC of the 10 randomly picked isolates (>128 mg/liter) after each passage in higher cefiderocol concentrations (Fig. 2a). Thus, confirming that cefiderocol resistance can develop under cefiderocol pressure in an *in vitro* experimental setting.

Because our clinical isolate, etcl_1, harbored two carbapenemase genes (*bla*_OXA-48_ and *bla*_NDM-5_), we repeated the experiment to investigate whether the presence of certain types of carbapenemases influenced the propensity for resistance development using different clinical E. cloacae isolates with similar genetic background (sequence type 96 by multilocus sequence type [MLST]). These were carrying other β-lactamases, namely, *bla*_OXA-48_ (KE9800), *bla*_KPC-2_ (KE9132), *bla*_NDM-5_ (KE5351), and a carbapenemase-negative type strain DSM30054 as a negative-control (Table S1). The growth of all E. cloacae isolates except for the one carrying a *bla*_NDM-5_ gene was inhibited during subculture with 1 mg/liter cefiderocol. Isolate KE5351 exhibited similar growth properties as the etcl_1 from our initial experiment, suggesting that the presence of NDM was associated with the development of cefiderocol resistance following cefiderocol exposure (Fig. 2a). Confirmatory experiments to determine the mutation frequency validated our initial findings. While DSM30054, KE9800, and KE9132 did not grow on cefiderocol-supplemented Mueller-Hinton agar at 4× and 10× the MIC of each isolate, both etcl_1 and KE5153 exhibited colony growth at both concentrations (Fig. 2b). Similar results were obtained for mutation experiments using Mueller-Hinton broth (liquid culture). Only etcl_1 and KE5351 exhibited growth at cefiderocol concentrations of 4× and 10× the MIC as determined by turbidity using a photometer at an optical density at 600 nm (OD_600_) after 8 and 24 h of incubation (Fig. 2c).

**FIG 3:**
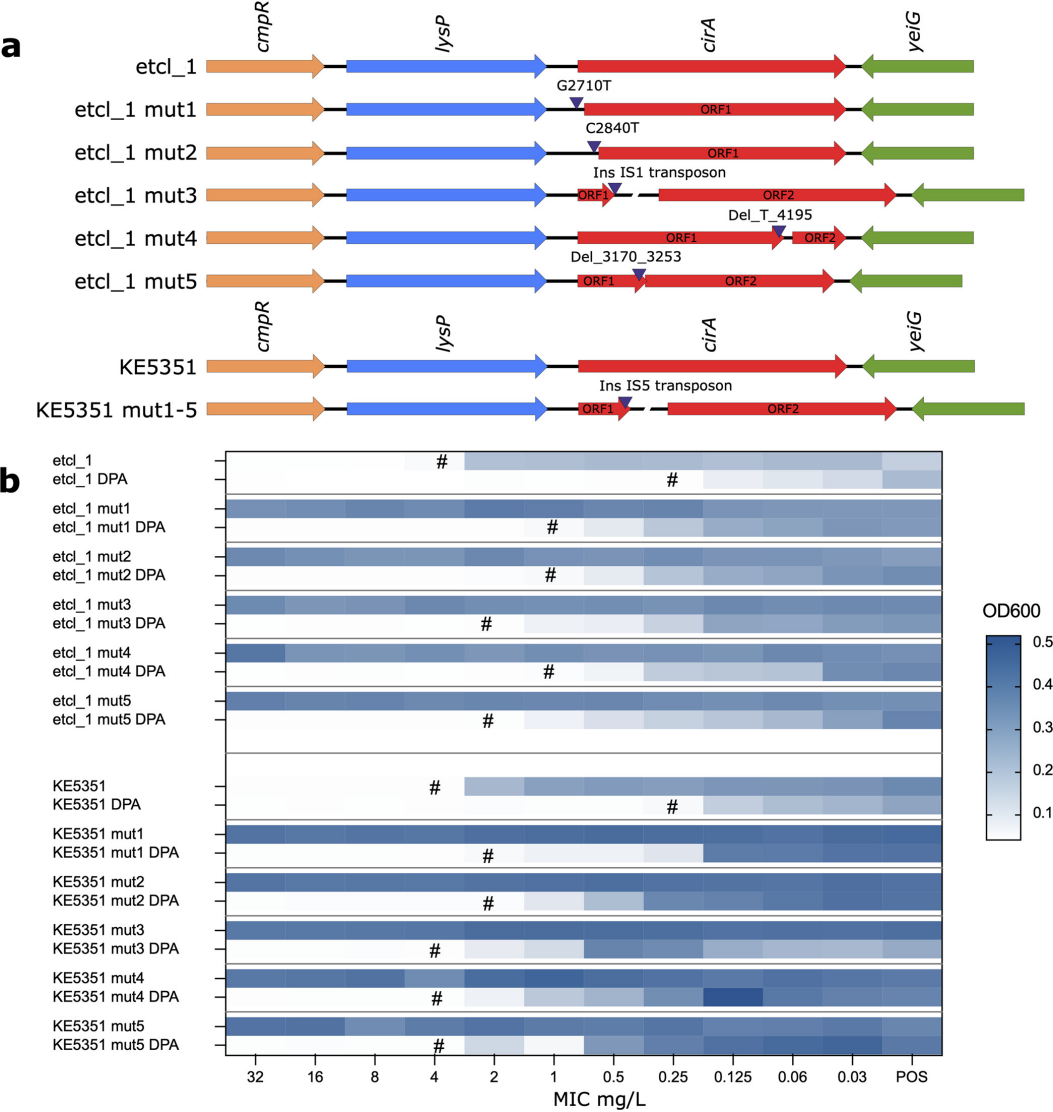
Heterogeneous mutations in the catecholate siderophore receptor gene *cirA* confer phenotypic resistance. (a) Alignment of the *cirA* gene between the cefiderocol-susceptible parental etcl_1 isolate and randomly picked resistant mutants (MIC >128 mg/liter) of etcl_1 (mut1 to mut5) revealed heterogeneous nonsynonymous alterations in the *cirA* gene of all resistant isolates. For the isolate KE5351, all resistant mutants (KE5351 mut1 to mut5) exhibited identical IS5-like transposon insertion leading to a truncation of the CirA protein. (b) Changes in the cefiderocol MIC associated with *cirA* mutations in the presence and absence of metallo-β-lactamase inhibitor, pyridine-2,6-dicarboxylic acid (DPA), measured at OD_600_ and displayed as a heat map. The acquisition of *cirA* mutation in the mutants generated by serial passaging led to an increase of the cefiderocol MIC from 4 mg/liter to >128 mg/liter (the highest concentration shown is 32 mg/liter). The cefiderocol MIC of isolates with *cirA* mutations increased from 0.25 mg/liter to 4 mg/liter independent of the metallo-β-lactamase activity. The MICs are indicated by “#”. The “POS” column indicates the growth control without antibiotics.

To investigate the underlying mechanism of resistance, we sequenced 5 of the 10 randomly picked isolates that grew at a cefiderocol concentration of 128 mg/liter from the serial passaging experiment for etlc_1 and KE5351 (Fig. 2a). Alignment of the core genome of the parental and the resistant mutant isolates (mut1 to mut5) revealed alterations in several genes (Fig. S2). However, only the catecholate siderophore receptor gene, *cirA*, and the IS5 family transposon IS903 were consistently mutated in cefiderocol-resistant derivatives of etcl_1 and KE5351. There were no alterations in the AmpC nor in *pbp3* genes, which have been attributed to reduced susceptibility toward cefiderocol in Enterobacterales (14, 15). All five resistant mutants of etcl_1 showed different nonsynonymous mutations in the *cirA* gene (two point mutations, two deletions, and one transposon insertion), suggesting a divergent evolution and confirming our previously reported *in vivo* finding (11). All resistant mutants of KE5351 were identical with an IS5-like transposon insertion on the *cirA* gene, possibly due to the expansion of a resistant clone (Fig. 3a). Genomic alterations or the acquisition of resistance may be linked by loss of fitness. To investigate whether the *cirA* mutations were associated with growth disadvantages for the bacteria, we performed a growth curve analysis in an optimal liquid medium (tryptic soy broth) and iron-depleted environment. In both conditions, there were no deviations in the growth characteristics of both isolates, indicating that the function of the siderophore catecholate receptor CirA may be redundant and that the loss of this function can be compensated by other mechanisms or other siderophore receptors (Fig. S1). Besides *cirA* mutation, cefiderocol resistance in Enterobacterales, specifically in Escherichia coli, has been attributed to the increased copy number and expression of the *bla*_NDM_ gene (16). Therefore, we investigated the abundance of the *bla*_NDM_ gene copy number using gene coverage from whole-genome sequencing (WGS) data normalized to the chromosomal *gyrB* as a proxy. For isolate etcl_1, there was no significant increase in coverage *bla*_NDM-5_ or *bla*_OXA-48_ in the cefiderocol-resistant mutants compared to the initial cefiderocol-susceptible parental isolate, suggesting no significant increase in the gene copy number. Similarly, for KE5351, there was also no significant increase in the sequencing coverage of *bla*_NDM-5_ compared to the initial isolate (Fig. S3). Furthermore, there was no significant increase in the mRNA expression of NDM (normalized to 16S rRNA) in the cefiderocol-resistant mutants compared to the initial cefiderocol-susceptible isolates (Fig. S3). Nonetheless, our data indicated that the production of NDM may have a partial effect on the phenotypic susceptibility toward cefiderocol.

To determine the net effect of *cirA* mutation on the MIC toward cefiderocol, we performed a broth microdilution for the initial and all cefiderocol-resistant isolates in the presence or absence of a metallo-β-lactamase inhibitor. For this purpose, we added 100 μg/mL dipicolinic acid (pyridine-2,6-dicarboxylic acid; DPA) into the iron-depleted CA-MHB. DPA is a chemical compound, which can chelate metal ions from NDM and, thus, inhibit its activity (17, 18). In the initial isolates, etcl_1 and KE5351, we observed a reduction of the MIC from 4 mg/liter to 0.25 mg/liter. In the isolates harboring NDM with a mutated *cirA*, the MIC was >128 mg/liter. However, in the presence of DPA, the MIC was reduced to 2 to 4 mg/liter (Fig. 3b), indicating that the acquisition of the *cirA* mutation led to an 8× MIC increase compared to the initial cefiderocol-susceptible isolates. Furthermore, our experiment suggested that the presence of NDM metallo-β-lactamase in the *cirA* mutants additionally and significantly reduced cefiderocol susceptibility, resulting in a high-level cefiderocol resistance.

**FIG 4:**
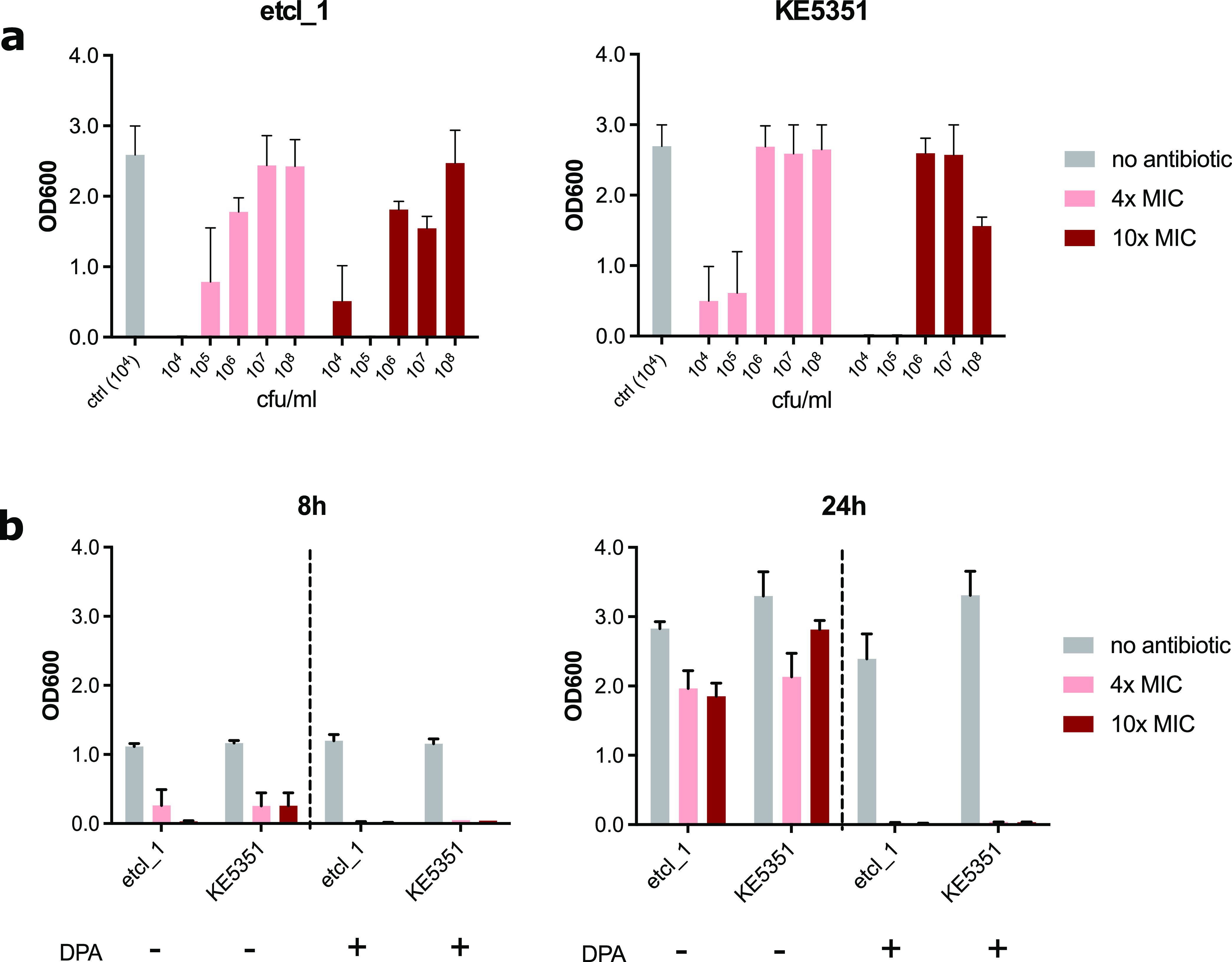
Inhibition of metallo-β-lactamase activity by pyridine-2,6-dicarboxylic acid prevents the emergence of cefiderocol resistance. (a) The influence of initial bacterial density (in CFU/mL) on bacterial growth under cefiderocol selection pressure (4× and 10× MIC of the respective isolate). Bacterial growth was quantified by a photometer (OD_600_). An initial inoculum of ≥10^6^ CFU/mL leads to bacterial growth under cefiderocol pressure consistently (in 3 out of 3 independent experiments). An initial inoculum of <10^6^ CFU/mL led to random emergence of cefiderocol resistance (1 out of 3 independent experiments from different inoculum). (b) The supplementation with 100 mg/liter pyridine-2,6-dicarboxylic acid (DPA) to inhibit the metallo-β-lactamase activity prevented the growth of resistant mutants under cefiderocol pressure.

For β-lactam antibiotics, the propensity for mutations and resistance development may be influenced by the inoculum effect. The inoculum effect is defined as attenuation of antibacterial activity at higher bacterial densities above those used for susceptibility testing (>5 × 10^5^ colony-forming unit [CFU]/mL) (19). This phenomenon is generally attributed to β-lactam antibiotics, especially cephalosporins, and has been recently described for cefiderocol (20). Higher bacterial density may increase the tolerant subpopulations and, thus, increase the chances of spontaneous mutations conferring resistance or leading to a more efficient enzymatic degradation of the antibiotic substance. To investigate whether the initial bacterial density influenced the resistance development, we repeated the resistance induction experiment using immediate antibiotic stress of 4× and 10× the MIC of the respective isolate. Bacterial growth (quantified photometrically at OD_600_) at cefiderocol concentrations of 4× and 10× the MIC can be consistently observed from an initial bacterial density of ≥10^6^ CFU/mL in all three independent experiments. For initial bacterial densities of 10^4^ and 10^5^ CFU/mL, resistant subpopulations emerged sporadically (1 out of 3 independent experiments from separate inoculums). Therefore, an inoculum effect may have a partial effect on the emergence of resistance. However, spontaneous mutations may also occur in lower bacterial densities, as suggested by our experiments (Fig. 4a).

Because the *cirA* mutations were only observed in metallo-β-lactamase-producing isolates, we then investigated if the inhibition of metallo-β-lactamase can prevent the emergence of resistant mutants. We repeated the experimental induction of cefiderocol resistance using 4× and 10× of the MIC for the isolates etcl_1 and KE5351 in the presence of DPA. By adding 100 μg/mL DPA into the liquid culture, we observed that bacterial growth was inhibited at 4× and 10× cefiderocol MIC compared to that of the DPA-free control (Fig. 4b). This observation suggested that the inhibition of NDM activity was sufficient to prevent the development of resistance.

## DISCUSSION

The presence of NDM facilitates the emergence of cefiderocol resistance in E. cloacae due to mutations in the *cirA* gene. In this study, the emergence of resistance phenotype was only observed in E. cloacae isolates harboring *bla*_NDM_ but not in carbapenem-susceptible pathogens or those harboring *bla*_OXA-48_. Although cefiderocol is stable against hydrolysis by metallo-β-lactamase, the elevation of the cefiderocol MIC within the susceptible range (cefiderocol tolerance) has been reported for NDM-type metallo-β-lactamase and may provide the necessary prerequisite for the acquisition of stable mutation conferring high-level resistance (21, 22). The reduction of the MIC from 2 to 4 mg/liter to <0.25 mg/liter for both etcl_1 and KE5351 in the presence of DPA supports this hypothesis. Indeed, other studies demonstrated that metallo-β-lactamases, such as IMP, VIM, and NDM, can marginally hydrolyze cefiderocol and lead to an increased MIC (7, 23). Consequently, the reduction of the cefiderocol concentration in our liquid culture may be reduced by this process, thus providing an ideal milieu to select for tolerant subpopulations and facilitate the development of stable resistance mutations.

Although cefiderocol resistance in Enterobacterales can still be considered rare, reports on the emergence of cefiderocol resistance Enterobacterales are cumulating (11, 16, 24). In Klebsiella pneumoniae and E. cloacae, the acquisition of cefiderocol resistance has been linked with mutations in the *cirA* gene, whereas, in E. coli, the emergence of cefiderocol resistance was associated with an increase in the *bla*_NDM-5_ copy number and gene expression. In our study, the *bla*_NDM-5_ gene copy number and expression were not responsible for the cefiderocol resistance phenotype. However, the presence of *bla*_NDM-5_ was at least partly responsible for the reduced susceptibility toward cefiderocol, as inhibition of the NDM-5 activity using DPA led to a decrease in cefiderocol MIC. Still, it did not entirely reduce the cefiderocol MIC to the baseline level so that both *cirA* mutation and NDM-5 activity may have an additive influence on the resistance phenotype. Of note, the emergence of cefiderocol resistance in Enterobacterales so far has been associated with the presence of *bla*_NDM-5_ on multiple occasions (11, 16, 24). Indeed, NDM-5 has been described to have increased hydrolysis activity toward β-lactam antibiotics than the NDM-1 variant (25). However, the role of the specific NDM variant in the propensity for the emergence of cefiderocol resistance is not yet elucidated and warrants further scrutiny.

Target gene inactivation via transposon insertion (also known as transposon-mediated insertional mutagenesis) (26) may explain the rapid emergence of cefiderocol resistance in metallo-β-lactamase-producing E. cloacae. Here, we demonstrated that, in 6 of the 10 sequenced isolates, the resistance phenotype was associated with an insertion of a transposable element. Furthermore, in the isolate with higher mutation frequencies, transposon insertion was found in all sequenced isolates. These *in vitro* findings are consistent with our previously published clinical case, where we identified an IS5-family transposon insertion in one of the resistant isolates. The idea of transposon insertional mutagenesis as a means of resistance acquisition is not completely novel but is not yet well investigated. Transposon insertional mutagenesis has been described to play an important role in contributing to high-level resistance toward ertapenem (27). In their experiments, Ma et al. (27) demonstrated that a reversible transposon insertion into the *ompK36* gene, encoding one of the major porins of Klebsiella pneumoniae, could be identified in ertapenem-resistant isolates. In contrast, in our case, the cefiderocol resistance was not reverted by serial passaging in the absence of antibiotic pressure, indicating the stability of the transposon insertion (data not shown). Fitness costs associated with the transposon insertion may explain this discrepancy. In the experiments of Ma et al. (27), the IS1 transposon insertion correlated with higher fitness costs such that, in the absence of antibiotic pressure, this insertion may be reverted to restore growth efficiency (27). In our case, the *cirA* disruption was not associated with an increased fitness cost and iron could be acquired via other means such that the mutation is not counter-selected. The preference of the insertion of a transposable element as a frequent resistance mechanism in E. cloacae suggests that translocation is a cost-effective option to acquire resistance and warrant further investigation.

Our findings have clinical implications. The treatment of infections due to metallo-β-lactamase-producing E. cloacae with cefiderocol needs close microbiological and clinical monitoring as cefiderocol-resistance can rapidly emerge during therapy (16). In particular, the emergence of resistance through transposon mobilization can jeopardize the efficacy of cefiderocol therapy for infections with carbapenemase-producing E. cloacae because the transposon-mediated disruption of the *cirA* gene in E. cloacae was not associated with impairment of the bacterial fitness. Our experimental study has some limitations. Both our clinical and laboratory findings are based on E. cloacae of the ST96 clonal group using a small number of isolates, and, therefore, further validation is needed. In this study, we chose to use isolates from the same clonal group to avoid bias due to lineage-associated sequence variations of *cirA* gene in E. cloacae. Other Enterobacterales or Gram-negative bacilli may be equipped with other or redundant catecholate siderophore receptors, which could delay the emergence of resistance. Furthermore, our data indicated that phenotypic resistance development may not be uniform at a given time, and, hence, the common practice of performing susceptibility testing on a representative isolate in the microbiological diagnostic may not accurately reflect the susceptibility of the whole bacterial population.

In conclusion, we demonstrated that the presence of the metallo-β-lactamase gene is a risk factor and facilitates the development of resistance toward cefiderocol in E. cloacae both *in vitro* and *in vivo*. The clinical use of this antibiotic substance in treating metallo-β-lactamase-producing Gram-negatives should be monitored closely for the emergence of resistance. On a positive note, the inhibition of metallo-β-lactamase activity could prevent the emergence of resistance. Further investigations are needed to study the potential benefit of combining this siderophore cephalosporin with β-lactamase inhibitors in preventing resistance development under therapy.

## MATERIALS AND METHODS

### Study isolates.

For this *in vitro* study, clinical E. cloacae isolates were selected based on their MLST (ST96) and the presence or absence of beta-lactamases (Table S1).

### Serial passage experiments.

To induce cefiderocol resistance, an experimental serial passaging under increasing cefiderocol selection pressure was performed. Briefly, a colony of E. cloacae growing on a Columbia agar plate with 5% sheep’s blood was cultured in 5 mL cation-adjusted Mueller-Hinton broth (CA-MHB) (BD Diagnostics, Germany) at 37°C under constant shaking at 200 rpm for 18 h. Following this, 100 μL of this overnight culture was transferred to a fresh 5 mL CA-MHB then cefiderocol (Shionogi, Japan) was added to reach a final concentration of 0.5 mg/liter and incubated in similar conditions to the initial culture. This process was repeated daily with increasing cefiderocol concentration by a factor of two until no visible turbidity/growth was identified after an overnight incubation or until the concentration of 128 mg/liter was reached. For the population analysis profile, 10 μL of the bacterial suspension from an overnight incubation was plated on Columbia blood agar, and 10 single colonies were picked at random for cefiderocol susceptibility testing by broth microdilution. In addition, disk diffusion using the liquid culture was performed to determine if a subpopulation has developed cefiderocol tolerance after each passage.

### Cefiderocol susceptibility testing.

Disk diffusion was performed with the standard Kirby-Bauer disk diffusion method according to the EUCAST guidelines. Briefly, 0.5 McFarland standard of a bacterial suspension in 0.9% NaCl was streaked in three different directions on Mueller-Hinton agar (bioMérieux GmbH, Germany), and 30 μg of cefiderocol disk (Liofilchem, Italy) was placed on the agar and incubated at 35 ± 1°C for 18 h. Cefiderocol MIC was determined by broth microdilution (range was 0.25 to 128 mg/liter) using iron-depleted CA-MHB, which was prepared according to the protocol by Hackel et al. (28), following CLSI and EUCAST recommendations for cefiderocol susceptibility testing. To determine the cefiderocol MIC without the influence of metallo-β-lactamases, a broth microdilution was performed using iron-depleted CA-MHB supplemented with 100 μg/mL pyridine-2,6-dicarboxylic acid (DPA) as a metallo-β-lactamases inhibitor. The MICs of the *cirA* mutants generated by serial passaging were determined using a cefiderocol concentration range of 0.03 to 32 mg/liter with and without DPA. The MIC was interpreted manually according to EUCAST broth microdilution interpretation guidelines and photometrically at OD_600_. MIC and disk diffusion were interpreted according to the EUCAST clinical breakpoints v.11.0.

### Growth curve analysis.

Growth analysis was performed on a 96-well microplate using iron-depleted cation-adjusted Mueller-Hinton broth and tryptic soy broth with an inoculum of 5 × 10^5^ CFU/mL in 150 μL total volume. The plate was incubated for 15 h at 37°C in a FLUOstar Optima (BMG Labtech) plate reader with shaking and the optical density at 600 nm (OD_600_) was measured every 5 min. Data were visualized using GraphPad Prism v.9 (GraphPad Software, USA), displaying the OD_600_ values every hour. The growth curve measurements were performed as biological and technical replicates.

### Whole-genome sequencing.

Genomic DNA was extracted from an overnight culture on Columbia blood agar using the DNeasy Blood and Tissue minikit (Qiagen, GmbH) following the manufacturer’s instructions. Library preparation and sequencing on a MiSeq Illumina platform (short-read sequencing 2 × 300 bp) were performed as previously described (11). Post sequencing procedure included quality control using sickle (v1.33; parameter “-q 30 -l 45”), assembly with SPAdes 3.13.0 (29) (with the options “–careful” and “–only-assembler”), and curation of the draft genome by removing contigs with a length < 1000 bp and/or coverage <10×. The quality of the final draft was quality controlled using Quast (v.5.0.2). To identify potential mutations associated with the development of cefiderocol tolerance, the draft genomes of the parental isolate were compared to the cefiderocol-resistant subpopulations using Mauve (30) and Snippy (https://github.com/tseemann/snippy).

### Gene comparison.

Genomes were annotated using Prokka v.1.14.0 (31). A core genome was calculated using Roary v.3.13.0 (32) and the core genome alignment was curated for recombination events with Gubbins v.3.1.3 (33) to build a phylogenetic tree comparing the resistant strain to the original strain. Each annotated genome from the resistant isolates was also compared to the original strain at the protein level to verify the impact of the nonsynonymous mutation. Each identical protein was clustered using CD-HIT (34) with an identity threshold of 100% and a coverage of 100%. The clustering results were transformed in the protein presence/absence matrix using in-house scripts.

### NDM coverage as a proxy for gene copy number.

Coverage of the gene *bla*_NDM-5_ and *gyrB* was calculated by mapping the curated fastq files to the gene sequence using Samtools (35). The coverage of *bla*_NDM-5_ was then normalized by the coverage of *gyrB* to ensure comparability between the isolates.

### NDM and OXA-48 mRNA expression.

RNA isolation and mRNA quantification was performed as described previously with minor modifications (36). Briefly, overnight cultures in iron-depleted CA-MHB were diluted 1:100 in fresh iron-depleted CA-MHB to a total volume of 2 mL and allowed to grow for 4 h to mid-log-phase. The bacterial suspension was then pelleted by centrifugation at 4000 rpm for 10 min. The supernatant was removed, and the bacterial pellet was harvested for RNA isolation. Total RNA was extracted using TRIzol and the Direct-zol™ RNA Miniprep kit (Zymo Research) according to the manufacturer’s protocol and followed by an additional DNase treatment using the RQ1 RNase-Free DNase kit (Promega). The expressions of *bla*_NDM_ and *bla*_OXA-48_ were determined by real-time quantitative PCR (RT-qPCR) (Luna Universal Probe One-Step RT–qPCR kit; New England Biolabs) relative to the 16S rRNA expression using previously published primers (37) for NDM-fw 5′-GCCACACCAGTGACAATATC-3′, NDM-rv 5′-GTGCTCAGTGTCGGCAT-3′, OXA-48-fw 5′-AGGGCGTAGTTGTGCTC-3′, OXA-48-rv 5′-GTGTTCATCCTTAACCACGC-3′‘and 16S-fw 5′-TCCTACGGGAGGCAGCAGT-3′, and 16S-rv 5′-GGACTACCAGGGTATCTAATCCTGTT-3′ in biological and technical duplicates.

### Determination of mutation frequency on solid culture medium.

Mutation frequency was determined by streaking bacterial isolates from overnight cultures on cation-adjusted Mueller-Hinton (CA-MHA) agar containing defined concentrations of cefiderocol (4× and 10× the MIC of the tested isolate). Briefly, a total of 100 μL of 0.5 McFarland standard of the bacterial suspensions (the equivalent of 1.5 × 10^8^ CFU/mL) were streaked on antibiotic-containing CA-MHA and on antibiotic-free CA-MHA to determine the overall viable population. The inoculated agar plates were incubated at 37°C for 18 h, followed by manual quantification of colony growth. The mutation frequency was defined as the number of counted colonies on the antibiotic-containing agar divided by the number of counted colonies on the antibiotic-free agar after 18 h of incubation.

### Photometric detection of resistance development.

To avoid selection bias due to random colony picking, we sought to investigate the emergence of resistance in liquid broth. Bacterial cells from an overnight culture were grown in fresh CA-MHB at 37°C under constant shaking (150 rpm) until the mid-log-phase was reached and was adjusted to 0.5 McFarland standard in fresh CA-MHB. This suspension (100 μL) was transferred to a fresh sterile culture tube containing 5 mL CA-MHB with (4× and 10× the MIC) without cefiderocol supplementation and incubated at 37°C under constant shaking at 150 rpm. Bacterial growth was quantified by measuring the turbidity at OD_600_ (Eppendorf, Germany) at 8 and 24 h using standard photometer cuvettes.

To investigate whether the inhibition of metallo-β-lactamase activity affected the emergence of resistance, an identical experimental set-up was applied with the addition of a known metallo-β-lactamase inhibitor, pyridine-2,6-dicarboxylic acid (DPA; dipicolinic acid) (Sigma-Aldrich, Germany), to reach a final concentration of 100 μg/mL in CA-MHB. Growth was defined as an OD_600_ that was greater than the OD_600_ of the medium-only control. All experiments were performed as triplicates in three independent experiments.

### Statistical analysis.

Statistical analysis and data visualization was performed using GraphPad Prism version 9 (GraphPad, USA). Wherever applicable, statistical significance was calculated using the Kruskal-Wallis test for nonnormally (nonparametric) distributed variables. A *P* value <0.05 was considered statistically significant (*, *P* < 0.05; **, *P* ≤ 0.01; ***, *P* ≤ 0.001).

### Data availability.

The draft genome sequences were deposited in the NCBI GenBank database under the project numbers PRJNA546126, PRJNA705064, and PRJNA750050. Sequencing statistics and isolate data are available in Supplemental Table S2.
