# Supplementary material for: New Delhi Metallo-Beta-Lactamase Facilitates the Emergence of Cefiderocol Resistance in Enterobacter cloacae
Source: Antimicrob Agents Chemother. 2022 Feb 15;66(2):e02011-21. doi: 10.1128/aac.02011-21 (PMC8846454; doi:10.1128/aac.02011-21)
Supplement: Supplemental file 1 — Supplemental material. Download aac.02011-21-s0001.pdf, PDF file, 0.6 MB [file aac.02011-21-s0001.pdf]

Supplementary Appendix

New Delhi metallo-beta-lactamase facilitates the emergence of cefiderocol resistance in *Enterobacter cloacae*.

Content

Supplementary Figure S1. Growth curve of Enterobacter cloacae with cirA mutations compared to the wild type. 2

Supplementary Figure S2. Comparison of gene protein sequence to identify functional mutations associated with cefiderocol resistance in *Enterobacter cloacae.* 3

Supplementary Figure S3. Sequencing coverage and expression of bla_{NDM} in cefiderocol-susceptible and cefiderocol-resistant *Enterobacter cloacae*. 4

Supplementary Table S1. Molecular characteristics of study isolates 5

Supplementary Table S2. Sequencing statistics 6

References....................7

Supplementary Figure S1. Growth curve of Enterobacter cloacae with cirA mutations compared to the wild type. There were no significant changes to the growth characteristics of *E. cloacae* isolates with mutated *cirA* gene compared to the initial wild type over 15 hours with measurements every 5 minutes. Data presented are measurements every 60 minutes as biological triplicates. a Growth curve for isolate etcl_1 in tryptic soy broth (TSB), left panel and in iron-depleted cation-adjusted Mueller-Hinton broth (ID-CAMHB), right panel. b Growth curve for isolate KE5351 in TSB (left panel) and ID-CAMHB (right panel).

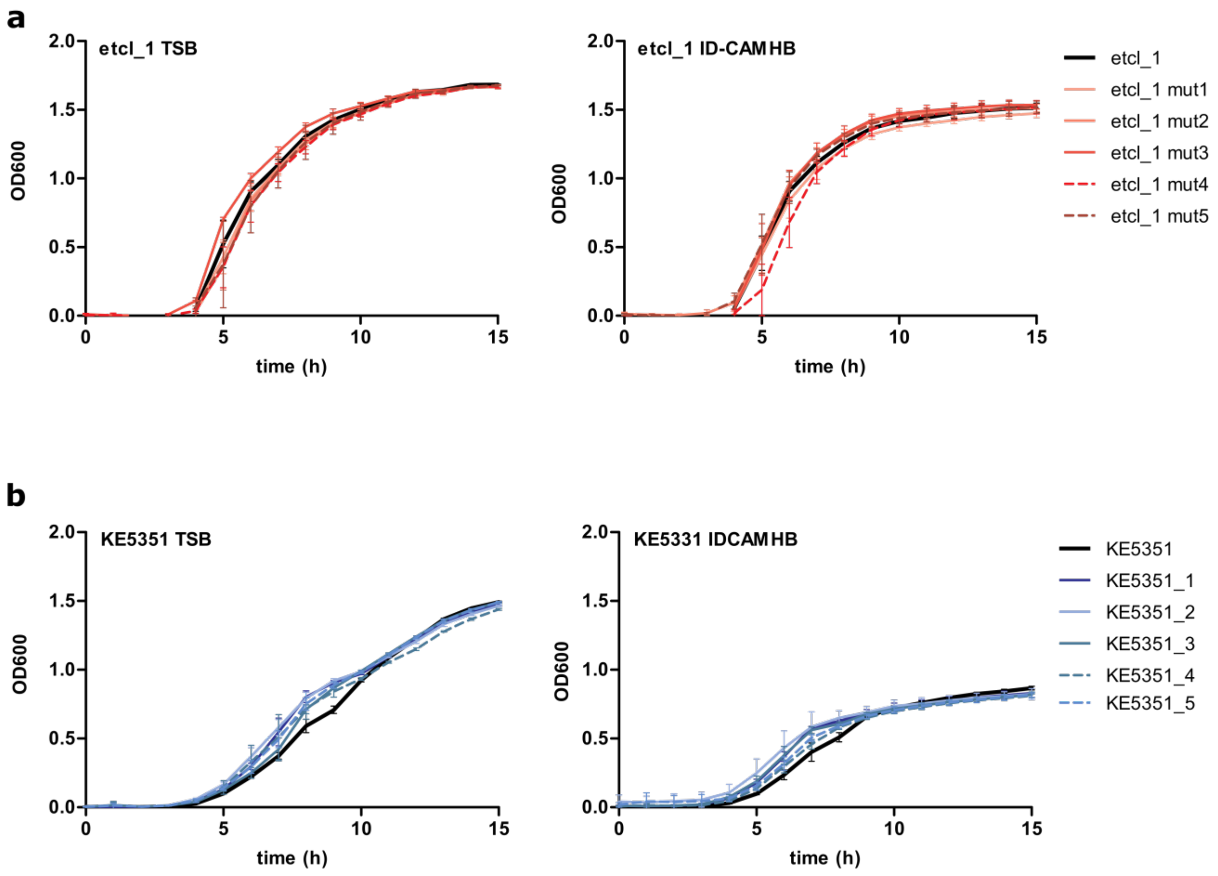

Supplementary Figure S2. Comparison of gene protein sequence to identify functional mutations associated with cefiderocol resistance in *Enterobacter cloacae.*

Gene-by-gene comparison (heatmap) at the protein level indicated that on the *cirA* gene and the IS5 family transposon IS903 were mutated in both in vitro generated cefiderocol-resistant mutants of etcl_1 (left panel) and KE5351 (right panel). Grey squares indicate non identical protein compared to the original strain; black squares indicate identical protein sequence to the wild type. The phylogenetic tree is based on the whole core genome curated for recombination event using Gubbins (4579638 nucleotides, 4815 genes for etcl_1 and 4554210 nucleotides, 4795 genes for KE5351.

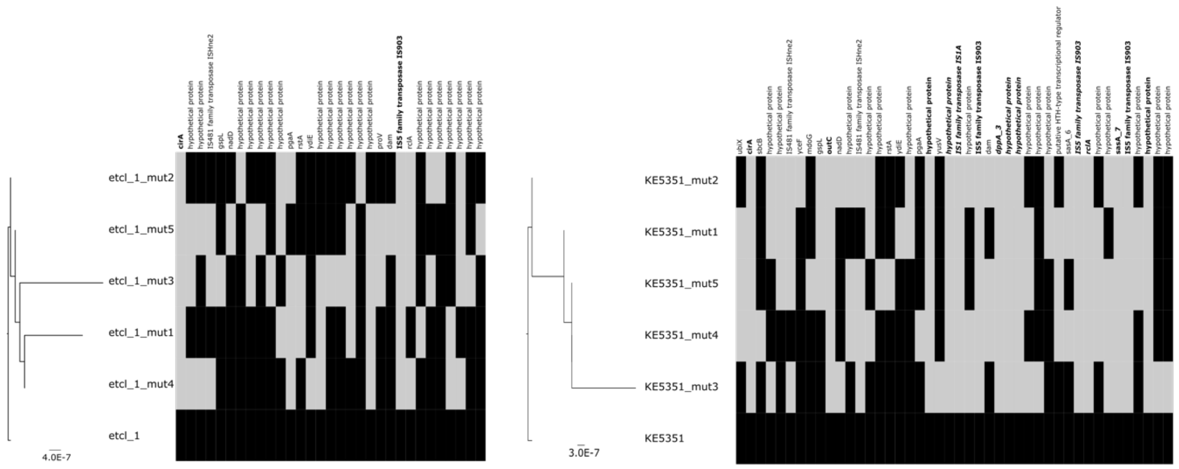

Supplementary Figure S3. Sequencing coverage and expression of *bla*$_{NDM}$ in cefiderocol-susceptible and cefiderocol-resistant *Enterobacter cloacae*.

(A) sequencing coverage of *bla*$_{NDM-5}$ (left panel) and *bla*$_{OXA-48}$ (right panel) relative to *gyrB* as a proxy for gene copy numbers. (B) mRNA expression of *bla*$_{NDM}$ relative to 16s *rRNA*, determined as biological and technical duplicates. There were no association between *bla*$_{NDM}$ expression or relative sequencing coverage and the phenotypic cefiderocol resistance, indicating that the cefiderocol resistance is not mediated by *bla*$_{NDM}$ overexpression.

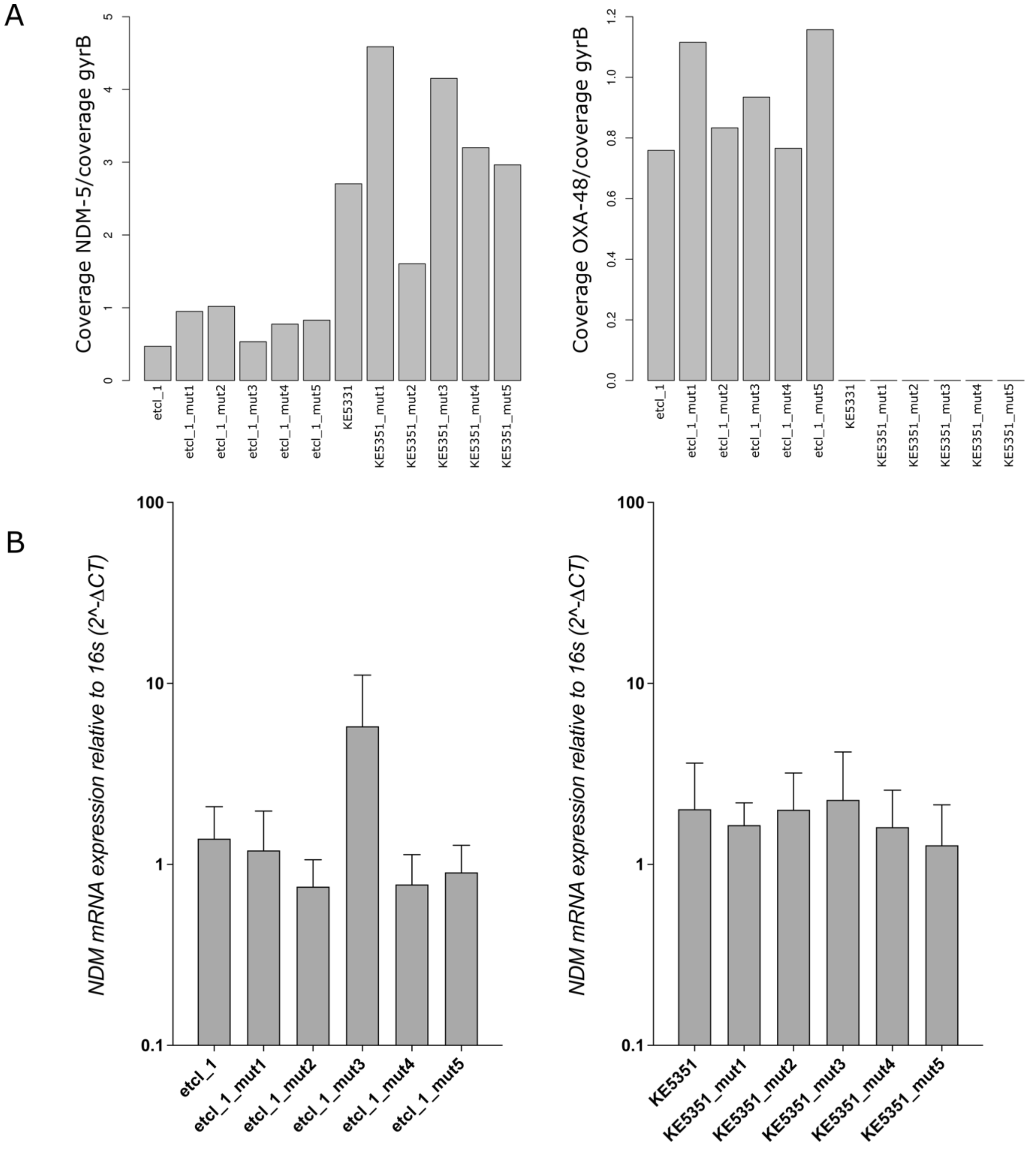

Supplementary Table S1. Molecular characteristics of study isolates

ID	Species	beta-lactamase	MLST	origin	study
DSM30054	*Enterobacter cloacae complex*	none	ST1	type strain	n/a
KE9800	*Enterobacter cloacae complex*	OXA-48	ST96	clinical isolate	this study
KE5351	*Enterobacter cloacae complex*	NDM-5	ST96	clinical isolate	this study
KE9132	*Enterobacter cloacae complex*	KPC-2	ST96	clinical isolate	this study
etcl_1	*Enterobacter cloacae complex*	NDM-5 and OXA-48	ST96	clinical isolate	Klein *et al.* [1]

Supplementary Table S2. Sequencing statistics

Sample ID	Species	MLST	Assembly SPAdes Version	Bioproject Number (NCBI)	Accession-Nr.	coverage	No. of contigs	Largest contig	Total length	GC (%)	N50	N75	L50	L75
etcl_1	*Enterobacter cloacae* complex	96	3.13.0	PRJNA705064	SAMN19988671	34	56	943666	5144974	55.38	269554	131255	5	12
etcl_1_mut1	*Enterobacter cloacae* complex	96	3.13.0	PRJNA750050	SAMN20428006	43	54	943666	5145625	55.38	269554	131255	5	12
etcl_1_mut2	*Enterobacter cloacae* complex	96	3.13.0	PRJNA750050	SAMN20428007	53	56	943666	5143992	55.39	269554	131255	5	12
etcl_1_mut3	*Enterobacter cloacae* complex	96	3.13.0	PRJNA750050	SAMN20428008	41	61	667504	5143401	55.38	250592	131255	7	15
etcl_1_mut4	*Enterobacter cloacae* complex	96	3.13.0	PRJNA750050	SAMN20428009	49	56	668231	5143938	55.38	269554	131255	6	13
etcl_1_mut5	*Enterobacter cloacae* complex	96	3.13.0	PRJNA750050	SAMN20428010	41	59	602811	5142507	55.38	269554	111882	6	14
KE5351	*Enterobacter cloacae* complex	96	3.13.0	PRJNA546126	SAMN21531226.	54	51	869925	5111164	55.39	322228	151054	5	11
KE5351_mut1	*Enterobacter cloacae* complex	96	3.13.0	PRJNA750050	SAMN20428011	42	57	667494	5105937	55.39	266038	131255	6	14
KE5351_mut2	*Enterobacter cloacae* complex	96	3.13.0	PRJNA750050	SAMN20428012	26	60	572807	5104811	55.39	198798	131255	8	15
KE5351_mut3	*Enterobacter cloacae* complex	96	3.13.0	PRJNA750050	SAMN20428013	31	55	667835	5103008	55.39	251756	131255	7	15
KE5351_mut4	*Enterobacter cloacae* complex	96	3.13.0	PRJNA750050	SAMN20428014	32	56	712929	5106387	55.39	251756	147562	6	13
KE5351_mut5	*Enterobacter cloacae* complex	96	3.13.0	PRJNA750050	SAMN20428015	39	57	667494	5107158	55.39	250587	131255	7	15

Abbrv. MLST=multi-locus sequence type

References

1 Klein, S. *et al.* Rapid development of cefiderocol resistance in carbapenem-resistant *Enterobacter cloacae* during therapy is associated with heterogeneous mutations in the catecholate siderophore receptor *cirA*. *Clin Infect Dis*, doi:10.1093/cid/ciab511 (2021).
